# A Comprehensive Review of Epigenetic Regulation of Vascular Smooth Muscle Cells During Development and Disease

**DOI:** 10.3390/biom16010173

**Published:** 2026-01-21

**Authors:** Lautaro Natali, Benjamín de la Cruz-Thea, Andrea Godino, Cecilia Conde, Victor I. Peinado, Melina M. Musri

**Affiliations:** 1 Mercedes and Martin Ferreyra Medical Research Institute, National Council for Scientific and Technical Research, National University of Córdoba (INIMEC-CONICET-UNC), Córdoba 5016, Argentina; lnatali@immf.uncor.edu (L.N.); agodino@immf.uncor.edu (A.G.); cconde@immf.uncor.edu (C.C.); 2 Department of Neuroscience, Cold Spring Harbor Laboratory, Cold Spring Harbor, New York, NY 11724, USA; delacruz@cshl.edu; 3 Department of Experimental Pathology, Institute of Biomedical Research of Barcelona (IIBB), Spanish National Research Council (CSIC), 08036 Barcelona, Spain; vpeinado@iibb.csic.es; 4 Department of Pulmonary Medicine, Hospital Clínic, Biomedical Research Institut August Pi i Sunyer (IDIBAPS), University of Barcelona, 08036 Barcelona, Spain; 5 Biomedical Research Networking Center in Respiratory Diseases (CIBERES), 28029 Madrid, Spain

**Keywords:** vascular smooth muscle cells, differentiation, vascular remodelling, cardiovascular diseases, epigenetics, RNA modifications

## Abstract

Vascular smooth muscle cells (VSMCs) in the tunica media are essential for maintaining the structure and function of the arterial wall. These cells regulate vascular tone and contribute to vasculogenesis and angiogenesis, particularly during development. Proper control of VSMC differentiation ensures the correct size and patterning of vessels. Dysregulation of VSMC behaviour in adulthood, however, is linked to serious cardiovascular diseases, including aortic aneurysm, coronary artery disease, atherosclerosis and pulmonary hypertension. VSMCs are characterised by their phenotypic plasticity, which is the capacity to transition from a contractile to a synthetic, dedifferentiated state in response to environmental cues. This phenotypic switch plays a central role in vascular remodelling, a process that drives the progression of many vascular pathologies. Epigenetic mechanisms, which are defined as heritable but reversible changes in gene expression that do not involve alterations to the DNA sequence, have emerged as key regulators of VSMC identity and behaviour. These mechanisms include DNA methylation, histone modifications, chromatin remodelling, non-coding RNA and RNA modifications. Understanding how these epigenetic processes influence VSMC plasticity is crucial to uncovering the molecular basis of vascular development and disease. This review explores the current understanding of VSMC biology, focusing on epigenetic regulation in health and pathology.

## 1. Introduction

Cardiovascular diseases (CVDs) are the leading cause of disease burden and mortality worldwide [1]. CVD encompasses a variety of conditions, including atherosclerosis, ischemic heart disease, ischemic stroke and cerebrovascular diseases, among others [1]. Although around 80% of CVD burden is attributable to modifiable risk factors, such as high systolic blood pressure, dietary risks, high levels of low-density lipoprotein cholesterol and air pollution [1], the widespread prevalence of these conditions underscores the importance of understanding the mechanisms underlying vascular homeostasis and their relationship to the development of CVD. Many of these non-communicable diseases involve a process known as vascular remodelling, whereby the structure of the vessel wall is altered in response to long-term changes in haemodynamic conditions [2,3].

In humans, blood vessels are distributed throughout the body in complex networks totalling over 100,000 km in length [4]. The structural characteristics of a blood vessel depend on its size and function. Large and medium-calibre veins and arteries consist of three layers or tunics: the adventitia (outer layer), which primarily consists of fibroblasts and a collagen matrix; the media (middle layer), which consists of concentric rings of vascular smooth muscle cells (VSMCs) and, in many arteries, elastic fibres; and the intima (inner layer), which consists mainly of a monolayer of endothelial cells (ECs) called the endothelium, along with its basal lamina [5]. During development, the size and pattern of the smooth muscle layer are precisely regulated in a vessel-specific manner. However, this regulation often becomes disrupted in major CVDs [6].

A substantial body of evidence demonstrates that VSMCs play a crucial role in maintaining the structure, integrity and function of the arterial wall [6,7]. VSMCs help stabilise the developing vessels during vasculogenesis (the process of de novo blood vessel formation) and during angiogenesis (the formation of new blood vessels from existing ones) [7]. Furthermore, VSMCs exhibit remarkable plasticity, enabling them to integrate diverse microenvironmental cues and modulate their behaviour accordingly, to participate actively in vascular repair processes [8,9]. However, this same VSMC plasticity also contributes to the pathogenesis of many CVDs [10].

Over the past few decades, epigenetics has emerged as a rapidly expanding field, particularly with regard to elucidating the mechanisms that govern cell fate determination during development and disease [11,12,13]. In this review, we provide an integrative overview of the phenotypic modulation of VSMCs under physiological conditions and in vascular pathologies, with a specific focus on the regulatory role of epigenetic mechanisms. Rather than providing an exhaustive summary of all the molecules involved in VSMC epigenetic regulation, we aim to emphasise the importance of the main epigenetic pathways, discuss their contribution to vascular homeostasis and disease and highlight the current challenges and therapeutic prospects in this evolving area of research.

## 2. Vascular Smooth Muscle Cells

### 2.1. Diversity of Embryological Origins and Phenotypic Plasticity

Smooth muscle cells (SMCs) are widely distributed across numerous organs, including the blood vessels, the trachea, the stomach, the small intestine, the corpus cavernosum and the uterus. They play a crucial role in the development and function of the cardiovascular, digestive, respiratory and urinary systems [14]. Here, we focus on VSMCs. Fully differentiated VSMCs typically have an elongated, spindle-shaped appearance with a heterochromatic nucleus and myofilaments, as well as fewer synthetic organelles [6]. VSMCs express a specific set of contractile proteins which they acquire during differentiation. The most abundant of these is smooth muscle α-actin (α-SMA), representing approximately 40% of total VSMC proteins and acting as an early differentiation marker, alongside calponin (CNN1) and transgelin (SM22α) [6]. Smooth muscle myosin heavy chain (SMMHC) [15] and smoothelin (SMTN) are expressed later in the differentiation process [16]. However, these markers are often not specific, since many of these proteins can also be found in other cell types, such as pericytes or fibroblasts [17], or in striated or cardiac muscle during development [18]. Among these markers, SMMHC appears to be the most specific marker of VSMCs [17]. An important characteristic of VSMCs is their tremendous diversity of molecular markers throughout the body, reflecting significant differences in function and regulation. This has largely resulted in complications in the study of VSMCs [19,20,21,22]. For instance, the lack of known VSMC-specific cell surface markers has hindered the isolation of specific VSMC populations for years, resulting in contradictory experimental results observed throughout the literature [22]. However, emerging technologies such as SMC lineage tracing and single-cell RNA sequencing have enabled researchers to gain a better understanding of SMC diversity in physiology and pathology [19,22,23] (see Section 2.4 below). This has led to the identification of new VSMC populations, intermediate cell subpopulations and novel molecular markers [19,22,24]. Recently, an inventory of murine VSMC markers was reported. The authors demonstrated the similarities and differences in the molecular signatures of SMCs in different organs, including arteries and veins [20]. Arterial SMCs display a far greater organotypic heterogeneity, whereas venous SMCs are more similar across organs [20]. Building on this research in humans, a comprehensive single-cell and spatial transcriptome atlas of multiple healthy arterial segments provided a detailed map of human vascular cellular identity [21]. This atlas revealed significant segment-specific cellular heterogeneity, predominantly within VSMCs and fibroblasts. Importantly, it confirmed that the variation in gene expression and cellular identity across the arterial tree is largely influenced by embryonic origin rather than anatomical location. Segment-specific differentially expressed genes were found to be enriched for genetic disease signals, which may explain the varying disease propensities (e.g., atherosclerosis and aneurysms) across different arterial segments [21].

**Figure 1:**
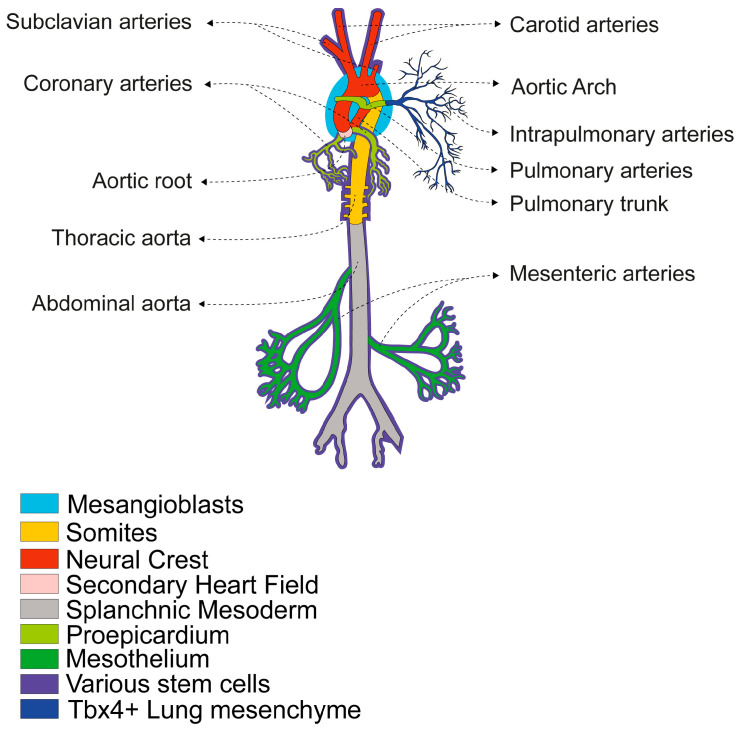
Developmental Origins of VSMCs in the Main Arteries. The diagram illustrates the different embryonic origins of VSMCs in different arteries, with a colour-coded key: Mesangioblasts (blue) give rise to intrapulmonary artery walls. Somites (orange) give rise to the descending aorta. The Neural Crest (red) contributes VSMCs to the major arteries near the heart, including the ascending aorta, the aortic arch, the pulmonary trunk, the ductus arteriosus, the aorticopulmonary septum, the innominate artery, the right subclavian arteries, the common carotid arteries and the arteries of the head and neck [25,31]. The Secondary Heart Field (pink) contributes VSMCs to the proximal great arteries, including the aortic root, the arterial pole at the base of the heart and the pulmonary trunk [31,32]. The Splanchnic Mesoderm (grey) gives rise to SMCs in the gastrointestinal and respiratory systems, as well as the systemic trunk and limb arteries, which contain most of the body’s musculature [14]. The Proepicardium (light green) is the source of progenitors for the coronary vessels [25,31]. The Mesothelium (dark green) gives rise to VSMCs within the mesenteric vasculature and its branches that penetrate the gut, as well as within pulmonary arteries [25,33]. Tbx4+ Lung mesenchyme (dark blue) gives rise to intrapulmonary artery walls [34] and various stem cells (violet) residing in the adult artery wall can differentiate into VSMCs or pericytes in response to growth factors or vascular injury [25,27].

One of the main reasons for this diversity is that the vascular system is a highly mosaic tissue, composed of various subtypes of VSMCs with different embryonic origins and characterised by specific signalling networks and cellular markers in each region [7,14,25,26,27,28]. VSMCs of different origins are found in different vessels and even within segments of the same vessel. The boundaries between VSMCs of different lineages are clearly defined, with cells of different origins rarely intermingling. Studies mainly using chick embryos and transgenic mice have produced a detailed map of the developmental origin of smooth muscle [14,25,26,27,28,29,30,31,32,33,34] (Figure 1).

These multiple origins reflect the diverse mechanisms involved in vascular development and influence how these cells respond to stimuli, injury, or disease in adulthood [25,35]. For example, aortic dissections frequently occur at points where VSMCs of different origins converge, potentially due to their distinct repair capacities [36,37]. These differences have led to the development of specific strategies for deriving distinct VSMC progenitors from human embryonic stem cells (ESCs) and induced pluripotent stem cells (iPSCs). There has also been an increase in the use of zebrafish for modelling vascular disease, drug discovery and vascular tissue engineering that take into account the heterogeneous embryonic origin [28,38,39].

**Figure 2:**
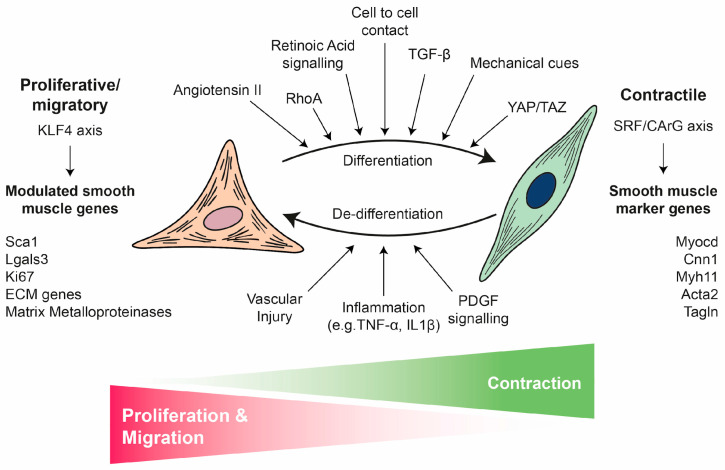
VSMC phenotypic switch: from contractile to proliferative/migratory phenotype. VSMCs exhibit remarkable phenotypic plasticity, switching between a differentiated, quiescent, contractile state (**right**) and a dedifferentiated, proliferative/migratory state (**left**). This switch is critical for vascular health and disease. The contractile VSMC phenotype (differentiated) is characterised by contraction and a low rate of proliferation and migration. This phenotype is maintained and induced by various cues including cell-to-cell contact, retinoic acid signalling, TGFβ, angiotensin II, RhoA, YAP/TAZ and mechanical cues. The expression of key smooth muscle markers (e.g., Myocd, Cnn1, Myh11, Acta2 and Tagln) is controlled by the SRF/CArG axis. Following stimuli such as vascular injury, inflammation (e.g., TNFα, IL1β), or PDGF signalling, VSMCs undergo a phenotypic switch to a proliferative/migratory VSMC phenotype (dedifferentiated). This is characterised by enhanced proliferation and migration as well as the expression of modulated genes (e.g., Sca1, Lgals3, Ki67, ECM genes and matrix metalloproteinases) which are mainly controlled by the KLF4 axis. This switch is essential for repair, but it also contributes to CVD.

Unlike skeletal and cardiac muscle, adult VSMCs retain remarkable plasticity both in vivo and in vitro, independently of their developmental origin [6]. They can transition between a differentiated and contractile phenotype to a dedifferentiated, synthetic, or proliferative phenotype in response to various environmental cues, such as growth factors, mechanical stress, cell-to-cell contact, cell–matrix interactions and inflammatory stimuli [6] (Figure 2). For example, when cultured in vitro, isolated mature VSMCs dedifferentiate and can then redifferentiate, particularly under conditions such as serum starvation or cell-to-cell contact [40,41,42,43]. This tightly regulated process, known as the phenotypic switch, is fundamental to vascular development and disease [6,8]. During early vascular development, VSMCs are typically proliferative and migratory and actively synthesise extracellular matrix (ECM) components. In contrast, mature VSMCs in the adult vasculature predominantly exhibit a contractile phenotype characterised by the expression of contractile proteins and minimal proliferation or migration [6,9]. Under pathological conditions such as CVD, VSMCs can revert to a synthetic state, downregulating contractile markers while increasing ECM synthesis and proliferation [6,9]. It is important to note that the boundaries between these phenotypes are not absolute, either during development [44] or in adult tissues [45].

### 2.2. Regulation of VSMC Phenotypic Switch

The differentiation and phenotypic plasticity of VSMCs are governed by an interplay of transcriptional networks, extracellular cues and mechanical stimuli [8,25,46,47,48]. Several in vitro and in vivo models have been established to study VSMC biology [49,50], and the mechanisms regulating VSMC differentiation and phenotypic transitions have been extensively reviewed [8,9,18,46].

Briefly, the central regulator of smooth-muscle-specific gene expression at the transcriptional level is the serum response factor (SRF), which binds to conserved CArG elements (CC(AT)6GG) within nearly all SMC-specific promoters to activate gene expression [6,51]. SRF activity is highly context-dependent, being modulated by post-transcriptional modifications, variation in CArG box affinity and spacing and interactions with multiple cofactors [51]. Among these cofactors, myocardin (MYOCD) and its related transcriptional regulators MRTF-A and MRTF-B act as potent SRF co-activators, enhancing SRF-CArG binding and inducing VSMC genes including SMMHC, calponin, α-SMA and SM22α [51]. MYOCD expression is induced by angiotensin II (Ang II), RhoA and transforming growth factor β (TGF-β) but is inhibited by inflammatory signalling through NF-κB, PDGF-BB and the insulin-like growth factor 1 (IGF1)-AKT-FoxO4 pathway [52]. Reduced MYOCD expression and/or activity is associated with VSMC dedifferentiation and has been observed in several models of vascular injury [52]. In contrast, Kruppel-like factor 4 (KLF4) functions as a major repressor of the contractile phenotype. It is rapidly induced in response to vascular injury, oxidative stress, or inflammation and suppresses VSMC-specific genes by disrupting the SRF-MYOCD axis and competing for promoter binding [47,53].

These transcriptional networks are modulated by multiple signalling pathways that converge on VSMCs. TGFβ signalling acts as a pivotal regulator by activating VSMC promoters through canonical Smad2/3-Smad4 complexes, while the non-canonical MAPK, RhoA/ROCK and PI3K/AKT pathways contribute to cytoskeletal remodelling and gene regulation [50]. Notch signalling is essential for vascular development and physiology [54]. It drives the differentiation of VSMCs into a contractile phenotype, but it also exerts context-dependent effects, promoting VSMC phenotypic switching and influencing survival, migration and ECM synthesis [54]. The Notch and TGFβ signalling pathways are closely related, acting synergistically or negatively regulating each other in specific contexts [54]. PDGF-BB also exhibits dual roles; it promotes the differentiation of mature VSMCs from progenitors but represses mature VSMC markers in adult cells via KLF4-mediated disruption of the SRF-MYOCD complex [47,50]. Retinoic acid (RA) signalling, via RAR/RXR heterodimers, promotes VSMC differentiation in vivo and in vitro by inducing PKCα, while also inhibiting KLF4 and enhancing VSMC gene expression [18,50,55]. Reactive oxygen species (ROS), particularly those generated by Nox4, are also essential for maintaining the differentiated phenotype [56]. For instance, Nox4-derived H_2_O_2_ enhances MYOCD-SRF complex formation through p38 MAPK signalling, whereas Nrf3 increases Nox4-mediated ROS production and promotes SRF binding to CArG boxes [57]. However, ROS can also mediate migration, apoptosis and the secretion of inflammatory cytokines and the ECM in VSMCs under pathological conditions [56]. The Wnt/β-catenin signalling pathway plays a role in VSMC biology by inducing the early specification and differentiation of VSMCs during development, as well as proliferation, migration and apoptosis in adult VSMCs [58].

In addition to biochemical regulation, the phenotype of VSMCs is strongly influenced by mechanical cues from the surrounding vascular environment. VSMCs are continuously subjected to circumferential stretch, shear stress and ECM stiffness [59]. These mechanical stimuli are physical and biochemical forces that are integrated through mechanotransduction and translated into intracellular responses via integrin-based focal adhesion (FA) complexes, which couple the ECM to the actin cytoskeleton [59]. These forces are then transmitted to the nucleus via the Linker of Nucleoskeleton and Cytoskeleton (LINC) complex, thereby enabling nuclear mechanosensing [60]. This mechanical signalling often converges on key effector pathways, most notably the Hippo pathway co-activators Yes-associated protein (YAP) and transcriptional co-activator with PDZ-binding motif (TAZ) [60]. YAP/TAZ then translocate to the nucleus and act as co-activators of TEAD transcription factors, thereby promoting MYOCD transcriptional activation [61,62]. Conversely, YAP/TAZ inactivation promotes the synthetic VSMC phenotype, vascular remodelling and inflammation [62]. Integrin activation triggers intracellular cascades involving FAK, PI3K and MAPK, which reinforce differentiation. Meanwhile, mechanosensitive ion channels, such as PIEZO1, TRP and BK, together with Notch and TGFβ receptor pathways, integrate physical and biochemical signals to fine-tune VSMC behaviour [63].

Taken together, these observations demonstrate that a multi-layered regulatory network, involving transcriptional, biochemical and mechanical inputs, acts on the MYOCD-SRF axis to maintain vascular homeostasis. Although extensive studies have revealed the primary drivers of VSMC homeostasis [8,9,10,46], the overall landscape of the pathways and factors that orchestrate VSMC phenotypic diversity during vascular repair and disease remains incompletely understood. Crucially, the signalling cascades detailed in this chapter are transient by nature. Consolidation of these signalling events into stable phenotypic changes requires epigenetic regulation, as discussed in Section 3.

### 2.3. The Role of VSMCs in Vascular Repair and Disease

**Figure 3:**
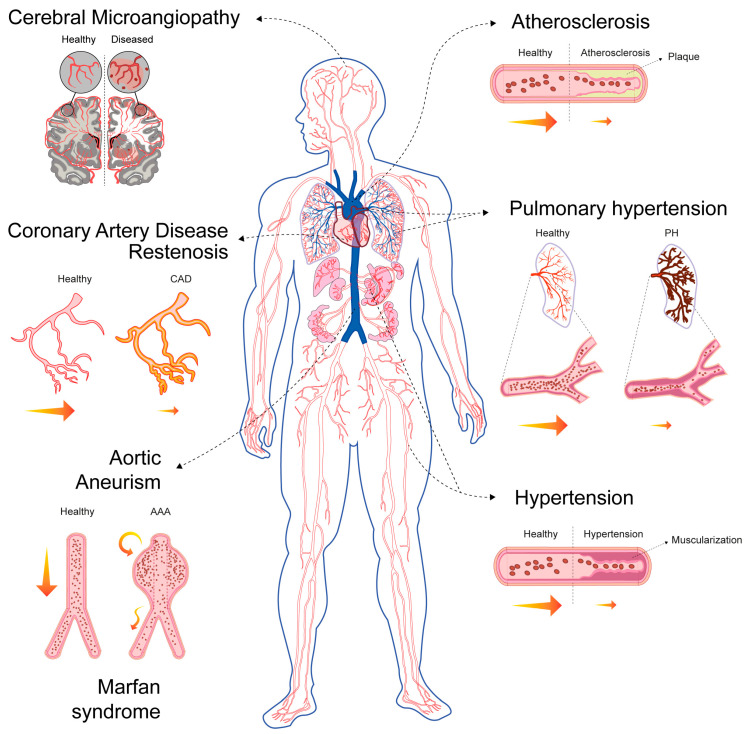
VSMCs in CVDs. This diagram highlights the central role of VSMCs in the pathophysiology of various CVDs. The diagram illustrates how VSMC dysfunction contributes to the following: Coronary artery disease (CAD) and restenosis: Healthy versus diseased coronary arteries are shown. Aortic aneurysm (AA): Localised enlargement of the aorta is depicted. Hypertension: Narrowed blood vessels in a hypertensive state compared to healthy vessels are illustrated. Cerebral microangiopathy: Small-vessel disease in the brain is shown, depicting hypoperfusion, lacunar infarcts (red areas) and microinfarcts (red spots). Atherosclerosis: The progression from a healthy vessel to plaque formation is shown. Pulmonary hypertension (PH): Constricted pulmonary arteries are displayed versus control arteries. Yellow arrows represent normal blood flow, decreased blood flow (small), turbulent blood flow (zigzag) or recirculation (circular).

Beyond their well-established roles in regulating haemodynamics and providing structural support, VSMCs are also critical to vascular repair processes [64]. The primary contributors to smooth muscle repair and vessel renewal are pre-existing, mature VSMCs, which possess a high degree of cellular plasticity [64]. In response to injury, these cells dedifferentiate to adopt reparative functions; once vessel homeostasis is restored, they redifferentiate into a mature, contractile phenotype. In healthy vascular walls, this dedifferentiation occurs at a very low rate to ensure continuous repair [64]. However, persistent and pathological VSMC-driven repair processes can contribute significantly to life-threatening conditions (Figure 3, Table 1). In this sense, pathological VSMC phenotypic modulation has been associated with tumour-cell-like behaviour and senescence, which are related to their inability to redifferentiate and the loss of plasticity [45,65].

Based on their specific roles in disease pathophysiology, VSMCs are involved in disorders characterised by three primary processes: excessive proliferation or intimal hyperplasia (e.g., atherosclerosis, restenosis, graft failure and pulmonary hypertension (PH)) [66], degeneration and/or apoptosis (e.g., abdominal aortic aneurysm (AAA) and Marfan syndrome) [67,68] or altered contractility (e.g., hypertension and cerebral microangiopathy) [69,70] (Figure 3).

Across the spectrum of vascular diseases, the pathogenic involvement of VSMCs hinges on a few interconnected mechanisms:

1. Pathological Phenotypic Modulation (Dedifferentiation): In pathological states, VSMCs suppress mature markers and activate genes associated with proliferation, migration and ECM synthesis [71,72,73,74]. This switch entails a loss of contractile function, and it can also involve transdifferentiation into other cell types, leading to mesenchymal, osteoblastic (calcification), or macrophage-like phenotypes, as observed in atherosclerosis [75,76,77]. Growth factors such as PDGF and pro-inflammatory signals such as IL-1 and TNFα are key drivers of this transition [45].
2. Proliferation and Migration (Neotintimal Hyperplasia): Synthetic VSMCs actively proliferate and migrate from their usual location in the tunica media into the innermost layer, the tunica intima [78]. This results in the thickening of the vessel wall and the formation of a new layer, the neointima [79]. Neointimal hyperplasia is the fundamental process responsible for lumen narrowing (stenosis), which leads to conditions such as restenosis, graft failure and PH.
3. Mechanotransduction and Metabolic Reprogramming: Mechanical stress is a key initiator. Disturbed flow and hypertension activate mechanoresponsive pathways in VSMCs. This stress promotes metabolic reprogramming [80]. Contractile VSMCs rely primarily on mitochondrial oxidative phosphorylation to supply the large amount of ATP needed for contraction [81]. Synthetic proliferative VSMCs undergo a metabolic shift often termed the “Warburg effect”, characterised by increased glucose uptake and dependence on glycolysis for energy even in the presence of oxygen [82,83,84]. This metabolic state supports rapid cell growth and proliferation, thereby directly contributing to vascular remodelling and PH [85].
4. Oxidative Stress and Mitochondrial Dysfunction: The metabolic shift is coupled with mitochondrial dysfunction [59]. Mitochondrial hyperfission and excessive ROS production stabilise HIF-1α, further enhancing the glycolytic programme in diseases like PH [56,84,86]. Chronic stress and metabolic dysfunction exacerbate vascular inflammation, which is a feature shared by many conditions, including atherosclerosis and aortic aneurysm (AA) [80,87].
5. Dual Role: In atherosclerosis, VSMCs play a critical dual role forming the protective fibrous cap contributing to plaque stabilisation [71,72]. However, their apoptosis, calcification, or dysfunction within the cap can lead to plaque rupture and thrombosis, which are the immediate cause of myocardial infarction and stroke [71].

Many vascular pathologies share overlapping features, highlighting the versatile and multifaceted nature of VSMC involvement in vascular diseases. However, these pathologies differ fundamentally in the dominant mechanisms of VSMC dysfunction (proliferation versus apoptosis), the affected vessel type and the final structural consequence (narrowing versus dilation) [66,67,68,69,70] (Table 1).

Despite the distinct aetiologies and clinical presentations of various vascular diseases, research and therapeutic development face several shared challenges. The most consistent and central challenge across all vascular pathologies is identifying the VSMC molecular signature in cells that have undergone de- or transdifferentiation during human disease progression. This is complicated by the fact that VSMCs rapidly lose their characteristic contractile markers during disease, which makes accurate identification, tracking and phenotyping of these cells within complex lesions extremely difficult, particularly in human tissue where lineage tracing is not yet possible [22,88,89]. Similarly, determining the true cellular origin of the proliferative lesions remains contentious, especially in human pathobiology [73,90,91], thereby complicating the precise targeting of pathogenic subsets. This lack of precise molecular understanding creates a significant therapeutic targeting dilemma: effective strategies must selectively inhibit the excessive and detrimental proliferative and migratory functions of VSMCs without simultaneously compromising their crucial protective roles, such as synthesising the collagen required for fibrous cap stability or maintaining normal vascular function [71,92,93].

**Table 1:** Comparative analysis of VSMC-driven mechanisms, structural consequences, and their specific contributions to the pathophysiology of major vascular diseases.

Disease and Affected Vessel	Dominant VSMC Mechanism	Structural Consequence	Contribution of VSMC to Pathophysiology
Atherosclerosis			
A chronic inflammatory condition that leads to endothelial dysfunction, gradual lumen narrowing and the formation of plaques (atheroma) in medium-to-large arteries (coronary, carotid, aorta) [94,95].	Excessive proliferation, migration and transdifferentiation into mesenchymal-like states, macrophage-like states, chondrocyte-like states, etc. [22,72,90].	Plaque formation (atheroma) in the intima, leading to stenosis and plaque instability that can lead to rupture and thrombosis [71,72].	VSMCs are central to plaque formation and progression, contributing 40–70% of plaque cells via medial VSMC migration and phenotypic modulation/transdifferentiation [22,77,96,97].
Pulmonary Hypertension (PH)			
A chronic, progressive condition characterised by a mean pulmonary arterial pressure (mPAP) of over 20 mmHg at rest and a pulmonary vascular resistance (PVR) of over 2.0 Wood units, as determined by right heart catheterisation. It affects small pulmonary arteries/arterioles [98].	Excessive proliferation and distal migration (muscularisation of arterioles) [99,100].	Pulmonary vascular remodelling (wall thickening, lumen narrowing) [101] formation of plexiform lesions [99,100,102,103].	Severe vascular remodelling caused by more than 90% of VSMC proliferation without evidence of transdifferentiation [73]. Arteriole muscularisation is driven by a pool of rare VSMC progenitors (PDGFR-β+, ACTA2+, MYH11+ cells) recapitulating key stages of arterial wall development [104,105,106].
Restenosis			
The recurrent narrowing (50% reduction in luminal diameter) of a blood vessel following revascularisation procedures. It affects stented/angioplastied arteries [107].	Excessive proliferation and migration [79].	Neointimal hyperplasia following mechanical injury leading to recurrent stenosis and graft occlusion [79].	A pathological wound healing response where VSMCs switch to a highly proliferative state in response to injury-induced growth factors [79]. Drug-eluting stents specifically target this VSMC proliferation [78,108,109].
Graft Failure			
Failure due to vascular grafts becoming occluded or stenotic following surgery. This affects vascular grafts, especially vein grafts [110]	Excessive proliferation (mid-term) and accelerated neoatherosclerosis (late-term) [111,112].	Intimal hyperplasia at the anastomosis site, followed by aggressive rupture-prone graft-specific atherosclerosia [112].	VSMCs are the primary source of cells that contribute to intimal hyperplasia. These cells assume multiple phenotypes similar to those observed in atherosclerosis [91].
Aortic Aneurysm (AA)			
A pathological localised enlargement of a segment of the aorta (abdominal or thoracic) [113].	Apoptosis and degeneration (VSMC loss) [67].	Progressive localised dilation and weakening of the aortic wall, increasing the risk of life-threatening rupture or dissection [114].	VSMC loss and reduced repair capacity are central, mediated by chronic inflammation and pro-apoptotic factors; VSMCs also exhibit a phenotypic switch, but loss is the dominant feature [115,116].
Marfan Syndrome (MFS)			
An autosomal dominant connective tissue disorder caused by mutations in the FBN1 gene, leading to multi-systemic defects, predominantly aortic root disease [117].	Apoptosis and degeneration (due to defective Fibrillin-1 (an ECM component) and excessive TGFβ signalling) [117,118].	Progressive aortic aneurysm and dissection (aortopathy) due to loss of structural integrity [117].	Medial degeneration and VSMC loss, compounded by phenotypic switch and ECM dysregulation (excessive collagen deposition) [119].
Hypertension			
A chronic disorder characterised by persistently high blood pressure, defined as >130/80 mmHg according to the ACC/AHA guidelines or >140/90 mmHg according to the WHO guidelines. It affects the systemic arterial network [120,121].	Altered contractility and increased mechanical wall stress [122].	Sustained elevated arterial pressure and increased risk of end-organ damage (e.g., stroke, kidney disease) [122,123].	Primarily involves an altered functional state of VSMCs, leading to chronic vasoconstriction and vascular remodelling [122,123].
Cerebral Microangiopathy			
A group of diseases that affect the small blood vessels in the brain, causing lesions and strokes. It can also lead to vascular cognitive impairment [124].	Altered contractility (remodelling/stiffening) and degeneration [125,126].	Small-vessel disease in the brain leading to white matter lesions, lacunar infarcts, and chronic hypoperfusion [127,128,129].	Structural remodelling characterised by phenotypic switch in VSMC degeneration. The combination of vessel stiffening and wall degradation is a key feature [125,126].

Closely related to the above, advancing clinical therapies is significantly hampered by the inability of current experimental systems to faithfully recapitulate the complexity of human vascular disease. In this sense, animal models often fail to perfectly mimic the full spectrum of human pathology, frequently reflecting acute disease processes rather than diseases’ chronic, progressive nature and a lack of key structural features found in human arteries [71,111,130,131,132]. For instance, VSMCs residing in normal human intima layers, which are not present in mice, might contribute significantly to intimal lesions [90]. Similarly, standard in vitro cell culture systems demonstrate critical deficiencies, as they struggle to replicate the native mechanical, haemodynamic, and immune microenvironment of the diseased vessel wall, thereby severely limiting the translational relevance of basic mechanistic findings [89,99]. Compounding these issues are technical constraints, such as the substantial barrier posed by the difficulty of studying challenging anatomical regions, like the distal arterioles in PH where vascular remodelling is most severe [99].

The underlying pathogenesis of vascular diseases involves intricate, nonlinear interactions between genetic, metabolic and mechanical factors, resulting in significant gaps in our fundamental understanding [71,99]. In this sense, altered mechanotransduction (disturbed flow, chronic mechanical stress and vessel stiffness) play a critical role as an initiating or perpetuating pathogenic event across multiple vascular diseases, including atherosclerosis, PH and hypertension, but it is often difficult to fully model and target [59,123,133]. Similarly, the cellular and molecular mechanisms that drive catastrophic events such as aneurysm rupture or plaque instability are not well understood, which makes it difficult to develop precise predictive markers [134,135,136].

Significant challenges persist in translating basic science into effective clinical tools and treatments, highlighting critical need for future research and development. The most urgent requirement is for reliable, non-invasive circulating biomarkers [136]. These biomarkers would enable clinicians to predict disease progression with greater accuracy, monitor plaque vulnerability more closely, assess the risk of aortic aneurysm rupture and track the efficacy of novel therapeutic agents in vivo. The current lack of such tools hinders early diagnosis and effective risk stratification [111,136,137]. Furthermore, a major unmet clinical goal for severe chronic vascular conditions is to develop of pharmaceutical agents that can safely and effectively reverse or induce the regression of advanced lesions or established vascular remodelling [92,135]. Current standard therapies often focus on slowing progression rather than achieving true resolution or reversal of the disease state [138,139,140]. Finally, therapeutic interventions are currently severely constrained by two technical issues: the difficulty of achieving efficient, localised drug delivery to the diseased vessel wall and the necessity of ensuring that only the pathogenic cells are targeted, without impairing the essential function of neighbouring healthy cells [136,141]. Addressing these constraints is crucial for minimising off-target effects and maximising therapeutic effectiveness.

### 2.4. The Impact of New Technologies on VSMC Research

As discussed in this review, a defining feature of VSMCs, particularly in disease contexts, is their remarkable phenotypic diversity. The development of bulk RNA sequencing (RNA-seq) in the early 2010s played a key role in characterising SMC phenotypes in vitro and identifying the signalling networks associated with them. However, this technique was fundamentally limited by the averaging of transcriptomic data across the entire sample, making it impossible to assess population-specific changes in gene expression in vivo.

Concurrently with the rise of bulk RNA-seq, lineage tracing studies enabled researchers to identify bona fide VSMCs in lesions [77,142]. These studies revealed that VSMCs could adopt extreme phenotypes, such as becoming osteochondrocyte- or macrophage-like cells [77,142]. Nevertheless, characterising these modulated VSMCs had relied on traditional panels of antibody markers, which lacked the resolution to capture the full spectrum of plasticity.

The introduction of single-cell RNA sequencing (scRNA-seq) provided the required level of resolution to overcome this limitation. When combined with lineage tracing, scRNA-seq became a transformative approach, enabling the analysis of gene expression profiles in thousands of individual cells [143] and revealing the full extent of VSMC heterogeneity and fate [22]. Armed with this new technology, researchers have characterised not only transcriptomic differences between VSMCs of different origins [20,21] but also differences within VSMC populations of the same origin [144]. This has led to the identification of cells in healthy tissues that already exhibit transcriptional signatures associated with disease states [145]. The discovery of these ‘preconditioned’ or ‘primed’ cells has had profound consequences for theories of VSMC involvement in pathogenic lesions [90], as described in Section 2.5.

This baseline heterogeneity is dramatically amplified in disease states, where distinct VSMC subpopulations coexist within the same lesion [53,146]. Pioneering scRNA-seq studies have confirmed that VSMC dedifferentiation and transdifferentiation are common features during disease progression, identifying different transcription factors as key drivers [145]. However, a key limitation of these studies is that scRNA-seq is a descriptive technique that only provides a snapshot of a cell’s transcriptomic state. While researchers can identify phenotypes, they can only infer the transcriptional trajectories that the cells took to reach them. To address this, Alencar et al., employed a sophisticated dual lineage tracking system [147]. They demonstrated that over 60% of plaque VSMCs transiently express galectin-3 (Lgals3), a marker that appears early during dedifferentiation downstream of KLF4, as they transition towards detrimental osteo- and mesenchymal-like phenotypes [147]. Notably, the previous sorting of lineage-traced VSMCs from late atherosclerotic lesions followed by scRNA-seq was also essential for resolving the heterogeneity of smooth-muscle-derived cell populations.

Another recently developed technique sits at the intersection between single-cell transcriptomics and epigenetics (or single-nucleus) sequencing for transposase-accessible chromatin (scATAC-seq). This technique identifies accessible or “open” chromatin at a resolution of a single cell [148]. When coupled with bioinformatic analysis, scATAC-seq enables for the identification of the transcription factors that drive specific phenotypes [149]. Furthermore, the analysis of chromatin regions that are accessible simultaneously enables researchers to map putative target genes affected by SNPs identified by GWAS and associated with CAD. These SNPs are often located in non-coding regions and affect gene expression by disrupting cell-specific enhancers [150]. Notably, most studies show a high degree of correlation in cell type annotation based on scRNAseq and scATAC-seq data, highlighting the critical relationship between chromatin accessibility and gene expression [150,151,152].

While scRNA-seq and scATAC-seq are excellent at identifying the cell subpopulations that drive pathologies, they lack information about the location of these cells. This is a critical gap in diseases with distinct structural heterogeneity, such as atherosclerosis. However, spatial transcriptomics combined with scRNAseq can now identify different subpopulations of cells expressing disease-relevant transcripts and map their location [153]. Although the field of spatial transcriptomics is still in its early stages, some laboratories are already applying this approach to cardiovascular disease [154,155,156]. In atherosclerosis, for example, this approach has allowed gene expression to be compared between the arterial wall, fibrous cap and necrotic core of the lesions pinpointing the location of senescent cells [156] and distinguishing between stable and unstable plaques [155]. A recent study utilising spatial transcriptomics found that in unstable plaques, CD68+ cells of true myeloid origin localise to the periphery or “shoulders” of the plaque, whereas CD68+ SMC-derived cells reside within the plaque [155]. While this aligns with previous reports of SMCs transdifferentiating into macrophage-like state in an atherosclerotic context [76,77,157], it also suggests that cells of myeloid origin occupy a different niche and may perform a different functional role to that previously thought.

Lastly, spatially resolved epigenomic techniques are also starting to emerge. Some of these techniques even allow for the simultaneous detection of RNA levels and chromatin accessibility [158]. Although these techniques have not yet been applied to smooth muscle research, they could be crucial in answering key questions in this area. For example, they could help to identify bona fide VSMCs in human pulmonary vascular remodelling lesions by their characteristic epigenetic signature at the MYH11 promoter. This signature is retained even after the loss of classical protein markers, as has already been identified in human atherosclerotic lesions [159] (see Section 3.2).

Despite their transformative potential, these novel techniques have limitations. These include a lack of robust sequencing depth and, in the case of spatial transcriptomics, the need to choose between probing a large panel of genes and achieving single-cell resolution [160]. Another limitation is the requirement for enzymatic dissociation in single-cell technologies, as not all cells respond equally to digestion, which can introduce bias [161]. Additionally, the high cost of these techniques makes working with large sample sizes difficult, which can lead to studies making generalised claims based on atypical samples.

Looking to the future, integrating single-cell proteomics will be essential for validating whether transcriptomic trajectories correspond to functional changes at the protein level. The continued use of dual lineage tracking systems, spatial analysis and conditional knockouts will ultimately be vital in identifying and targeting the transcriptional regulators that promote the transdifferentiation of VSMCs into harmful phenotypes.

### 2.5. The Oligoclonal Origin of SMCs in Disease

For the last half century, a central question in the study of atherosclerosis has been whether the cellular mass of a plaque arises from the stochastic proliferation of local cells or the clonal expansion of a select few. The first direct evidence of the clonal origin of atherosclerotic lesions was presented by Benditt and Benditt in 1973 [162]. By analysing tissue from women who were heterozygous for the X-linked glucose-6-phosphate dehydrogenase (G6PD) gene, they made the surprising observation that atherosclerotic plaques predominantly exhibited only one G6PD isoform, whereas the surrounding tissue exhibited both isoforms [162]. This led to the ‘monoclonal hypothesis’, which proposes that plaques originate from a single transformed cell, drawing a parallel with tumour formation [163].

Over the decades, the current consensus that plaque origin is oligoclonal has emerged [164,165,166]. Modern studies using multi-colour lineage tracing of transgenic mice have revealed that the oligoclonal expansion of a small subset of VSMCs is a defining characteristic not only of atherosclerosis but also of other vascular pathologies involving VSMC phenotypic switching. These pathologies include pulmonary arterial hypertension (PAH) [74], vascular injury [165,166,167] and aneurysm formation [168].

Several mechanisms have been proposed to explain this oligoclonality. One prominent hypothesis suggests the existence of a ‘primed’ subpopulation of VSMCs that responds excessively to pro-proliferative environmental signals. Evidence for this theory comes from the identification of such primed cells in distal arterioles [105,169] and pulmonary arteries [74]. A subset of VSMCs that express stem cell antigen 1 (Sca-1, which is encoded by the Ly6a gene) has been identified as a potential primed progenitor population that is prone to clonal expansion [145]. However, the role of Sca1 as a marker of a primed VSMC population has been challenged. Single-cell transcriptomic assays consistently reveal a continuous gradient of VSMC dedifferentiation in disease states, contradicting the notion of a discrete, pre-existing primed population [144]. Furthermore, Sca1 has been successfully induced in VSMCs by promoting their dedifferentiation in vitro, suggesting that Sca1 may be a marker acquired by cells undergoing dedifferentiation [144]. Additionally, the translational potential of this marker is limited by the absence of a direct human Ly6a homologue.

While the search for a primed progenitor yields contradictory results, the oligoclonality of VSMCs in lesions remains a robust observation. Consequently, alternative or complementary explanations are still being put forward [19,170,171]. For example, it has been proposed that the challenge of crossing the internal elastic lamina to reach the intima could restrict the number of progenitors responsible for intimal hyperplasia [172]. Drawing a parallel with cancer, it has also been proposed that clonal expansion results from a subset of VSMCs developing immune evasion by expressing the “don’t eat me” molecule CD47, which enables them to grow unchecked [173]. It is likely that many of these mechanisms exist simultaneously, promoting oligoclonality to a varied extent.

Many exciting questions remain unanswered: Does a true marker for primed VSMCs in the major arteries exist? Would manipulating this specific cell population lead to less serious lesions, or just lesions with a smaller VSMC contribution? Would preventing VSMC clonal proliferation interfere with physiological wound healing?

The alteration in cell fate during the pathological phenotypic switch of VSMCs is the direct result of underlying epigenetic mechanisms, which translate external, pathological cues into stable, heritable changes in gene expression [45,174]. These processes actively modulate gene expression, thereby reprograming the VSMC transcriptome and locking the cell into its new phenotype [175]. In the following sections, we will explore the various known epigenetic mechanisms that govern VSMC gene expression and its alterations in pathological contexts.

## 3. Epigenetics

The concept of epigenetics is rooted in the pioneering work of embryologist Conrad Waddington, who proposed that an organism emerges from a single unit during development (a process known as epigenesis), utilising its genetic information [176]. He emphasised the important contributions of genes and environmental factors to the specialisation of cell types and tissues during development. Subsequently, epigenetic mechanisms were discovered that connect environmental influences with gene expression and cell fate decisions, maintaining these connections during cell division and ensuring their heritability [177]. According to Wu and Morris, epigenetics was therefore defined in 2001 as the study of changes in gene function that are mitotically and/or meiotically heritable without altering the DNA sequence [178]. However, the precise definition of epigenetics remains the subject of some debate [179,180].

**Figure 4:**
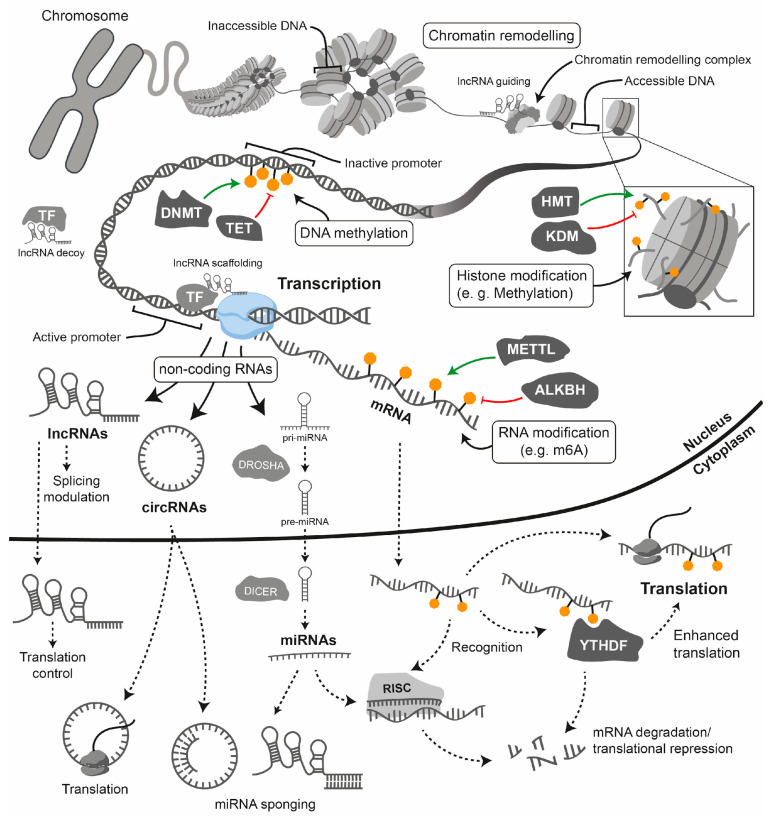
Epigenetic and post-transcriptional gene regulation. This simplified diagram illustrates the key epigenetic and post-transcriptional mechanisms that control gene expression, from the structure of chromatin in the nucleus to the translation of mRNA in the cytoplasm. The chromosome (**top left**) depicts the highly condensed state that is characteristic of mitosis.

For a gene to be transcribed, the DNA must be in a relaxed, or ‘open’, configuration that allows access for transcription factors and the transcriptional machinery [181]. This accessible state is typically found at the promoters and enhancers of actively expressed genes [181]. Epigenetic processes modulate the structural and functional state of chromatin, converting it from a compact, transcriptionally repressive state (heterochromatin) to a relaxed, transcriptionally permissive state (euchromatin) and vice versa, thereby regulating gene expression. For our purposes, therefore, we use the term ‘epigenetics’ to refer to the mechanisms that regulate gene expression independently of changes in the DNA sequence [181]. Figure 4 depicts the core epigenetic mechanisms, which include DNA methylation, post-translational histone protein modifications, chromatin remodelling and non-coding RNAs [181]. Although distinct from classical epigenetics, epitranscriptomics is increasingly recognised as part of the broader epigenetic regulatory landscape [182]. Epitranscriptomics refers to the study of biochemical modifications to RNA molecules that regulate their function [183]. However, there is ongoing discussion as to whether epitranscriptomics should be considered part of epigenetics, since epitranscriptomic marks affect expression, but they are not necessarily inherited during mitosis or meiosis [182,184].

**Figure 5:**
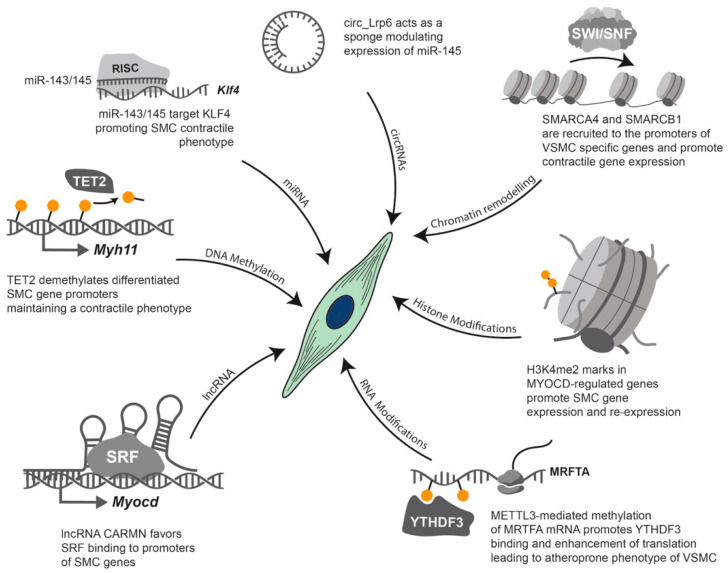
Epigenetic regulation of VSMC phenotype. This diagram illustrates examples of the key epigenetic and epitranscriptomic mechanisms that regulate the VSMC phenotype, which plays a central role in CVDs. Various molecular pathways converge on VSMCs, modulating their contractile versus synthetic state. DNA demethylation: TET2 demethylates specific promoters (e.g., Myh11), to maintain a differentiated, contractile VSMC phenotype. miRNA regulation: miR-143/145 directly targets *KLF4* mRNA via the RISC, to promote a contractile VSMC phenotype. Circular (circRNA) sponging: circ_LRP6 acts as a sponge for miR-145, promoting a dedifferentiated VSMC phenotype. Histone modifications: Specific histone modifications, such as the demethylation of lysine 4 in histone 3 (H3K4me2) on MYOCD-regulated genes, promote VSMC gene expression and re-expression, thereby influencing the VSMC phenotype. RNA modifications (e.g., m6A): METTL3-mediated methylation of MRTFA mRNA promotes the binding of the “reader” protein YTHDF3. This enhances the translation of MRTFA, contributing to a synthetic phenotype in VSMCs. Long non-coding RNAs (lncRNAs): *CARMN*, a lncRNA, favours the binding of the serum response factor (SRF) to SMC promoters, such as the MYOCD promoter.

Epigenetic modifications can be stable and heritable through cell divisions, thereby maintaining cell identity and lineage-specific patterns of gene expression. However, they are also dynamic, enabling cells to respond to developmental and environmental signals such as diet, smoking, alcohol consumption, stress and physical activity [174]. This dynamic regulation plays a pivotal role in embryogenesis, cellular differentiation and pathological conditions such as CVD and cancer [174]. Importantly, aberrant epigenetic modifications are increasingly recognised as key features of various diseases, highlighting their potential as therapeutic targets [175] (Figure 5). Therefore, understanding the epigenetic regulation of gene expression is essential to elucidating the complex mechanisms underlying normal development and disease progression.

Transcriptional regulation: Gene regulation begins with chromatin structure: the inaccessibility of DNA within heterochromatin is due to its tight association with histones. Chromatin remodelling complexes move nucleosomes to create accessible DNA for the transcriptional machinery (euchromatin). Histone modifications: The inset illustrates a nucleosome with modified histone tails (e.g., methylation), which regulate transcription. Enzymes such as histone methyltransferases (HMTs) deposit these modifications, while histone demethylases (KDMs) erase them. DNA methylation: This modification is triggered by DNA methyltransferases (DNMTs) and reversed by TET enzymes. It typically leads to transcriptional repression when it occurs at gene promoters. Long non-coding RNAs (lncRNAs) participate in transcriptional regulation by acting as guides for chromatin remodelling complexes, as decoys for transcription factors, by scaffolding chromatin complexes or by directly affecting splicing.

Post-transcriptional regulation: Transcription by RNA polymerase II generates mRNA and various non-coding RNAs (ncRNAs), including microRNAs (miRNAs), long non-coding RNAs (lncRNAs) and circular RNAs (circRNAs). RNA modifications (e.g., N6-methyladenosine or m6A): Modifications such as m6A are incorporated co-transcriptionally by complexes containing METTL3 and are removed by erasers such as ALKBH5. Methylated mRNAs are recognised by YTHDF “reader” proteins, which direct them towards either enhanced translation or degradation. miRNA processing and function: Primary miRNAs (pri-miRNAs) are first processed by Drosha in the nucleus and then by Dicer in the cytoplasm. The mature miRNA is then loaded into the RNA-induced silencing complex (RISC), which leads to the inhibition of translation and the degradation of target mRNAs. lncRNAs and circRNAs have diverse functions: lncRNAs can serve as scaffolds or bind to mRNAs to act as miRNA sponges, while circRNAs can also act as miRNA sponges or be translated into peptides.

### 3.1. DNA Methylation

DNA methylation, which primarily involves the generation of 5-methylcytosine (5mC) at CpG dinucleotides, is a well-studied epigenetic mechanism [185,186]. This modification is catalysed by DNA methyltransferases (DNMTs): DNMT3A and DNMT3B carry out de novo methylation, while DNMT1 maintains the pattern of DNA methylation during replication [187,188]. Consistently with its dynamic nature, 5mC can be demethylated via passive or active processes. Passive demethylation involves DNA synthesis without methylation, so 5mC becomes diluted during cell division [189,190,191]. Active demethylation involves ten-eleven translocation (TET) enzymes, which oxidise 5mC to form hydroxymethylcytosine (5hmC), formylcytosine (5fC) and carboxylcytosine (5caC) [190]. Subsequently, 5fC and 5caC can be removed by thymine DNA glycosylase in a process coupled with base excision repair [190]. Deformylation of 5fC is an additional mechanism that restores unmodified cytosine [192].

One of the most recognised functions of DNA methylation is the suppression of transcriptional activity [193]. Methylated cytosines can hinder the binding of transcription factors [194] or recruit and bind methyl-CpG-binding domain (MBD) proteins such as the methyl-CpG-binding protein 2 (MeCP2) [195].

Research shows that a balanced activity of DNA methylation enzymes is essential for maintaining the differentiated state of healthy VSMCs. Disruption to this balance is a hallmark of vascular disease [46,196]. For example, inhibiting DNMTs can prevent VSMC dedifferentiation and proliferation, thereby mitigating vascular remodelling [197]. Conversely, TET2 is crucial for preserving the VSMC contractile phenotype; its repression, which is frequently observed in vascular disease, decreases the protective 5hmC enrichment on the promoters of differentiation marker genes and reduces expression of genes associated with contraction [198,199].

DNA methylation provides a stable epigenetic memory that links the VSMC phenotype to environmental and mechanical cues. Mechanotransduction can inhibit DNMT1, driving VSMCs towards an osteogenic phenotype via the discoidin domain receptor 1 (DDR1)/extracellular-signal-regulated kinase (ERK)/p53 pathway [200,201,202]. Furthermore, DNA methylation, the VSMC phenotypic state and the cellular metabolic state are tightly coupled [203]. S-adenosylmethionine (SAM), a universal methyl donor and essential DNMT substrate, influences global methylation [203]. Similarly, α-ketoglutarate (α-KG), a TCA cycle intermediate, acts as a critical cofactor for TET enzymes, thereby modulating the VSMC phenotype [203,204]. Additionally, reactive oxygen species (ROS) can directly modulate epigenetic enzymes: moderate ROS levels activate TET2, promoting 5hmC formation on contractile gene promoters. In contrast, chronic oxidative stress inactivates TET2, leading to decreased 5hmC levels and the silencing of differentiation genes [205].

Despite its regulatory power, the therapeutic application of this mechanism is limited by two key challenges: the lack of specificity in current pharmacological modulators, which poses a high risk of systemic toxicity when managing chronic diseases [203], and persistent mechanistic gaps regarding the functional significance of non-CpG methylation and the precise roles of all TET oxidation products in diseased VSMCs. Future efforts must therefore focus on developing highly cell- and locus-specific targeting strategies, whether through advanced delivery systems, highly selective small molecules or proteolysis-targeting chimaeras (PROTACs), in order to safely exploit this epigenetic mechanism and restore a healthy VSMC state (see Section 4).

### 3.2. Histone Modifications

Eukaryotic DNA is organised into nucleosomes, which are composed of DNA wrapped around an octamer of core histones: H2A, H2B, H3 and H4 [206]. The N-terminal tails of these histones are targets for numerous post-translational modifications, including acetylation and methylation, which are fundamental to the regulation of gene expression [207]. Histone acetylation catalysed by histone acetyltransferases (HATs) adds an acetyl group to lysine residues, particularly lysines 9 and 27 of histone 3 (H3K9 and H3K27, respectively) [208]. This neutralises the positive charge of the lysine residue, resulting in an open, relaxed euchromatin structure that facilitates transcription and promotes robust gene expression [208,209]. Conversely, histone deacetylases (HDACs) remove these acetyl groups, restoring the positive charge and leading to a condensed heterochromatin structure that represses gene expression [210]. Histone methylation, catalysed by histone methyltransferases (HMTs) and removed by histone demethylases (HDMs), has context-dependent effects [211]. Specific marks define regulatory regions: monomethylation of histone 3 lysine 4 (H3K4me1) marks enhancers, and H3K4me2 marks active promoters and enhancers [11,159,212]. H3K4me3 marks active promoters [213], while H3K9me3 and H3K27me3 are well-known repressive marks [214].

During development, VSMCs acquire a unique histone modification signature that is essential for establishing and maintaining their specialised contractile phenotype [11,215]. This is achieved through histone acetylation in promoter regions of contractile protein-harbouring gene promoters (such as sm22α, Myh11 and Acta2) early in VSMC differentiation, which establishes an accessible chromatin state [216,217]. Additionally, H3K4me2-specific enrichment is evident in the promoters of VSMC marker genes in mature VSMCs and their committed progenitor cells, highlighting its specificity to the smooth muscle lineage [159]. Anatomical differences in VSMCs, reflecting their distinct embryonic origins, are associated with varied gene expression and an epigenetic profile [218]. Differences in chromatin accessibility among vascular sites may act as epigenetic memory, potentially leading to differential responses during vascular disease [218].

Pathological phenotypic switching is often driven by signalling pathways that recruit HDAC (e.g., HDAC2, HDAC4 and HDAC5) via factors such as KLF4. This leads to the deacetylation and repression of contractility-associated genes, as well as the enrichment of repressive marks such as H3K9me3 [219].

Thus, maintaining the differentiated state requires a delicate balance between HAT and HDAC, as well as HMT and HDM, activity, which is disrupted in pathological conditions [11]. Specific HDAC isoforms, including HDAC1, 2 and 3 [220] and HDAC9 [221], exhibit increased activity in vascular disease models, resulting in widespread deacetylation and loss of the contractile phenotype. While HAT p300 promotes the contractile phenotype by enhancing acetylation and cooperating with TET2 [222,223], HAT CBP can promote dedifferentiation into a proliferative state [224]. Additionally, mechanosensitive co-activators such as YAP/TAZ recruit p300/CBP and BRD4 to regulate gene expression in response to mechanical cues [225].

The sirtuin (SIRT) family of NAD^+^ deacetylases, particularly SIRT1, are critical regulators of vascular health and remodelling [226]. As metabolic sensors, their activity depends directly on the intracellular NAD^+^ level, which declines with ageing and metabolic stress. Reduced NAD^+^ inhibits SIRT activity, resulting in histone hyperacetylation and the repression of genes encoding contractile proteins, such as MYH11 and ACTA2 [226].

Despite the proven role of HDACs and their inhibitors in treating other diseases [227] and the fact that HDAC inhibitors have been approved for use in treating other conditions, none are currently used for CVD [226,228]. The main obstacle to progress is the systemic toxicity and off-target effects associated with broad-spectrum inhibitors, given the fundamental role of these enzymes in all cell types [226,227]. Future progress critically hinges on two critical avenues. Firstly, achieving isoform-specific and cell-targeted modulation, such as developing of specific HDAC or SIRT modulators coupled with localised delivery to the lesion. Secondly, a deeper mechanistic understanding is needed of the context-dependent function of individual marks and the precise mechanisms by which metabolic cues (e.g., NAD^+^ and acetyl-CoA) regulate these key epigenetic writers and erasers in VSMCs [226].

### 3.3. Chromatin Remodelling

Chromatin remodelling is an energy-dependent process involving the reorganisation or replacement of nucleosomes. It is critical for modulating the accessibility of transcriptional machinery to gene regulatory regions [229]. This process is essential for VSMC differentiation and function [230]. Key chromatin remodellers in VSMC biology are the SWI/SNF (or BAF) complexes, which utilise core ATPase subunits, primarily SMARCA4 (Brg1) or SMARCA2 (Brm) [231]. These complexes interact with various factors to exert gene-specific effects. For example, SMARCA4 interacts with MYOCD to regulate contractile protein expression [232], and its presence is necessary for the induction of VSMC-specific genes [233]. However, the role of SMARCA4 in disease is context-dependent and complex. While knocking out SMARCA4 causes severe cardiovascular abnormalities [234], increased expression of the protein is paradoxically observed in human aortic dissection and vascular injury models, where inhibiting SMARCA4 improves vascular remodelling and reduces inflammation [235,236]. Pathologically, SMARCA4 can collaborate with repressive factors such as HDAC9 and EZH2 to silence contractility-associated genes [237], and both SMARCA4 and SMARCA2 are required to activate inflammatory genes in response to mediators such as endothelin-1 (ET-1) [238]. SMARCD proteins are also vital [230]. SMARCD3 is essential for the differentiation of VSMCs acting as a co-activator for SRF to promote contractile protein expression [239]. However, its expression is significantly decreased in AAA samples, promoting VSMC apoptosis [240,241,242]. Conversely, SMARCD1 is increased in VSMCs in AAA, promoting inflammation [243], and its inhibition blocks proliferation and migration [244]. Given the proven role of ATP-dependent chromatin remodellers in regulating VSMC fate and pathology, the therapeutic modulation of these proteins is a rapidly evolving area, particularly in oncology [245]. However, to date, no pre-clinical or clinical studies have focused on testing the potential therapeutic role of these molecules in CVD.

### 3.4. Non-Coding RNAs

Since the sequencing of the human genome, a great deal of attention has been given to genes that encode translatable RNA, including mRNA and coding RNA. However, these represent less than 2% of the human genome [246]. By contrast, the remaining 98% of non-coding regions have been dismissed as ‘junk DNA’ [247]. This significant disparity underscores the importance of non-coding sequences. Indeed, a correlation has been observed between the complexity of an organism and the ratio of coding to non-coding genes, emphasising their regulatory significance [248,249]. The transcribed RNA from these genes can generally be divided into two groups: ‘housekeeping ncRNAs’, which fulfil general functions, and ‘regulatory ncRNAs’, which have regulatory functions [250]. The first group has been the focus of extensive research and comprises mainly ribosomal RNAs (rRNAs) and transfer RNAs (tRNAs), which are primarily involved in protein synthesis. It also comprises small nuclear RNAs (snRNAs), which participate in intron excision and alternative splicing, and small nucleolar RNAs (snoRNAs), which deposit modifications in other housekeeping RNAs [250]. There are also RNAs involved in telomere maintenance, known as “telomerase RNAs” [250]. The second large group (regulatory ncRNAs) comprises two subgroups: long non-coding RNAs (lncRNAs) and small non-coding RNAs (sncRNAs), which are defined as being above or below 500 nucleotides (nt) in length, respectively [251]. The sncRNAs can be divided into three main groups: microRNAs (miRNAs), small interfering RNAs (siRNAs) and Piwi-interacting RNAs (piRNAs), as well as a numerous novel types of sncRNA that do not fit into the established classes [252]. Several groups of non-coding RNAs can be classified as in either the long or small group due to their highly variable length. These include promoter-associated transcripts (PATs), enhancer RNAs (eRNAs) and circRNAs [253]. Therefore, Mattick et al. [251] suggested classifying ncRNAs into three categories: (1) sncRNAs (less than 50 nucleotides (nt)); (2) RNA polymerase III (Pol III) transcripts (e.g., tRNAs, 5S ribosomal RNA (rRNA), 7SK RNA, 7SL RNA and Alu RNA, Vault RNA and Y RNA) and Pol V transcripts in plants and small Pol II transcripts (e.g., most snRNAs and intron-derived snoRNAs); (3) lncRNAs (more than 500 nucleotides (nt)), which are mostly generated by Pol II.

#### 3.4.1. Small Non-Coding RNAs

Since the beginning of the century, substantial progress has been made in the field of sncRNAs, particularly microRNAs. These are sncRNAs that play a crucial role in regulating gene expression, with over 2000 estimated to influence more than 60% of protein-coding genes [254,255].

Canonical miRNA biogenesis begins with transcription into pri-miRNAs, followed by nuclear cleavage by the Drosha/Drosha-related (Drosha/DGR8) complex into pre-miRNAs and cytoplasmic processing by Dicer into mature duplexes [256]. These mature transcripts are generally 21–25 nt long, and the guide strand is incorporated into the RNA-induced silencing complex (RISC), which contains Argonaute (AGO) [256].

MiRNAs typically bind to the 3’ untranslated region (UTR) of target mRNAs, leading to translational repression and/or mRNA degradation [256]. Extracellular or circulating miRNAs are recognised as crucial mediators of cell-to-cell communication, existing either in a vesicle-associated form (e.g., exosomes) or in a protein-bound form (e.g., with AGO2 or HDL) in various biological fluids [257].

The essential role of microRNAs in VSMC differentiation and cardiovascular development has been confirmed in Dicer knockout models [258,259]. Numerous miRNAs modulate the VSMC phenotype and influence processes such as proliferation, migration, inflammation, and calcification. They are also consistently associated with vascular pathologies [9,42,260,261]. The fate of VSMCs is dynamically influenced by the microenvironment. Mechanical cues such as shear stress, cyclic stretch and stiffness, ROS and metabolic conditions modulate key miRNAs [201,261,262]. A wide range of miRNAs have been implicated in VSMC biology and proposed as potential biomarkers for various vascular conditions, including atherosclerosis [263], CAD, restenosis [264], PH [265], aneurysms [266] and hypertension [267], among others [261]. Key miRNAs that modulate VSMC phenotype include the miR-143/145 cluster, which promotes the contractile phenotype by targeting *KLF4* [268,269]. Deletion of this cluster in SMCs impairs the acquisition of the contractile phenotype and is associated with vascular diseases [270,271,272]. Other miRNAs, such as miR-133, inhibit the synthetic phenotype [273], while the miR-221/222 cluster promotes proliferation [274,275]. Conversely, the miR-29 family exhibits potent anti-fibrotic effects and promotes VSMC differentiation [276,277,278]. Our laboratory has found that, in patients with chronic obstructive pulmonary disease (COPD), the expression of miR-98, miR-139-5p, miR-146-5p and miR-451 was significantly higher in pulmonary arteries than in those of non-smoking controls [42]. Among these, the expression of miR-197 was found to correlate with the degree of both airflow obstruction and pulmonary artery thickening. In vitro, miR-197 expression was associated with a contractile phenotype, and inhibition of miR-197 promoted a proliferative phenotype by targeting *E2F1* [42].

Despite the demonstrable regulatory power of miRNAs and their promising preclinical therapeutic potential [279,280], such as the silencing of miR-145 in PAH [272], two major challenges must be overcome for clinical success. The first is the need for safe and effective targeted delivery systems to ensure therapeutic stability and action within the specific diseased vascular cell type [261]. Secondly, the functional specificity of extracellular miRNAs must be elucidated, as must the mechanisms by which they are packaged, secreted and taken up by recipient cells to mediate long-range communication [257,281].

Future research must focus on overcoming these delivery barriers by leveraging advances in nanomedicine and engineered extracellular vesicles [282] to safely translate the undeniable regulatory power of sncRNAs into viable therapeutic strategies for CVD.

#### 3.4.2. Long Non-Coding RNAs

The current consensus is that lncRNAs exceed 500 nt, with the 200–500 nt range being considered ambiguous [251]. LncRNAs are crucial, multifaceted regulators of gene expression with highly temporal and spatial specificity [251,283]. They perform a plethora of functions in both the cytoplasm and the nucleus [251]. In the cytoplasm, they can regulate the stability and translation of mRNAs, serve as bridges for multiprotein complexes, stabilise protein–RNA interactions, act as miRNA “sponges”, facilitate protein modifications, regulate the nucleocytoplasmic transport of transcription factors and even harbour the coding of small peptides (micropeptides) [251]. In the nucleus, they regulate transcription by guiding or sequestering transcription factors and/or chromatin remodellers to specific chromosomal sites. They also act as enhancer RNAs and participate in the alternative splicing of pre-mRNAs. Furthermore, they regulate nuclear organisation by forming biocondensates [251]. LncRNAs modulate enzymatic activity linking them to metabolic pathways [284]. Hundreds of thousands of lncRNAs have been catalogued, though this number remains an underestimate [285,286]. LncRNAs are critical, multifaceted regulators of gene expression that play pivotal roles in various stages of organism development, differentiation and cell fate determination [283]. LncRNAs participate in several steps of cardiovascular development [287] and are integral regulators of VSMC phenotype and function during development and disease [9,260,288,289].

Recent single-cell and spatial transcriptomic studies have emphasised the importance of lncRNAs in determining regional vascular identity and disease risk in humans [21]. Segment-specific lncRNAs are significantly enriched for genetic signals associated with diseases such as atherosclerosis and AA [21]. This suggests that lncRNAs are not only general regulators of VSMC phenotype but also crucial determinants of the spatial-specific susceptibility to pathology in the adult human vasculature. Specific lncRNAs influence the pathological conversion to an osteogenic or chondrogenic phenotype. For example, *H19* accelerates vascular calcification and modulates VSMC function in aortic dissection [290,291], while *ALIVEC* promotes chondrogenic differentiation and contributes to vascular stiffness [292]. Other examples, such as *CASC2* and *VELRP*, regulate VSMC proliferation in pulmonary vascular disease [293,294]. LncRNAs often act as competing endogenous RNAs (ceRNAs) by sponging miRNAs, as in the case of *H19* sponging miR-106a-5p [290,291], or they regulate contractile gene expression via signalling pathways such as the Akt/mTOR and Notch pathways, for example, *FOXC2-AS* and *NR21-AS1* [295].

**Figure 6:**
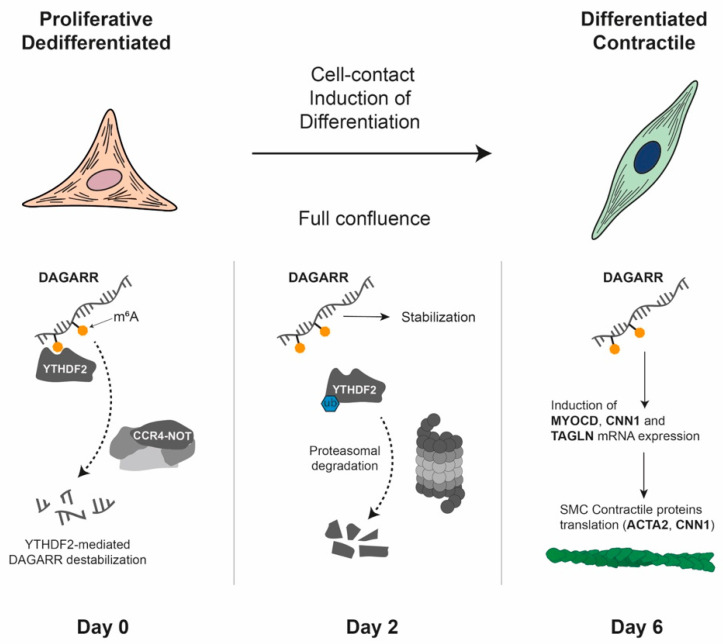
Proposed mechanism of *DAGARR*-mediated smooth muscle cell differentiation. The schematic illustrates the phenotypic transition of vascular smooth muscle cells (VSMCs) from a proliferative/dedifferentiated state to a differentiated/contractile state following contact inhibition. On day 0 (70% confluence), the m6A reader protein YTHDF2 binds to m6A-modified sites on the *DAGARR* and recruits the CCR4–NOT deadenylation complex, promoting *DAGARR* destabilisation and degradation. On day 2 (100% confluence), upon induction of differentiation by cell-to-cell contact, YTHDF2 undergoes proteasomal degradation. The resulting decrease in YTHDF2 protein levels allows *DAGARR* transcript stabilisation, leading to a marked expression increase. By day 6 (4 days post-confluence), stabilised *DAGARR* promotes the expression of contractile marker genes (*MYOCD*, *CNN1*, *TAGLN*), the synthesis of VSMC contractile proteins (such as ACTA2) and the formation of stress fibres.

Our laboratory has recently described a novel lncRNA that we named DAGARR (Differentiation- And Growth-Arrest-Related lncRNA) [296]. This naming reflects the inverse relationship between its expression and cell proliferation, while at the same being necessary for proper VSMC differentiation induced by cell-to-cell contact (Figure 6). We found this lncRNA is also downregulated upon VSMC stimulation with pro-inflammatory factors such as TNF, and similarly, its knockdown promotes proliferation and hinders the expression of differentiation and contractile-cytoskeleton genes [296]. One of the key findings upon *DAGARR* knockdown was the downregulation of MYOCD, suggesting a very upstream role in VMSC phenotypic modulation. Surprisingly, *DAGARR* was found to regulate growth in human fibroblasts, suggesting a broader biological function in maintaining tissue homeostasis and preventing aberrant fibroproliferative responses. We identified *DAGARR* as a new marker of cellular quiescence and found that its expression is significantly downregulated in the pulmonary arteries of patients with chronic obstructive pulmonary disease (COPD). Mass spectrometry analysis of *DAGARR* pulldown revealed that it binds several key proteins known to control VSMC plasticity, including Rho GTPase regulators and, notably, Transferrin receptor 1 (TFRC) [296]. Additionally, we observed the association of *DAGARR* with the N6-methyladenosine (m6A) methylation machinery and its associated proteins, a lesser-known epigenetic mechanism detailed in Section 3.5. RNA pulldowns using an anti-m6A antibody revealed that the *DAGARR* transcript is m6A-modified, consistently with methylation motifs found in *DAGARR* sequence, and further experiments confirmed the regulation of *DAGARR* stability via a YTHDF2-dependant mechanism. The proteasomal degradation of YTHDF2 upon cell-to-cell contact allows *DAGARR* transcript stabilisation and further induction of the VSMC contractility-associated transcriptional plan [296]. However, characterising the precise functional consequences of all protein interactions observed for this transcript remains difficult, and its full molecular mechanism should be further explored. This fact reflects a recurrent obstacle in the field, since lncRNAs are very much dependent on the cellular context.

The current challenge in lncRNA research is the insufficient mechanistic understanding of their function. These molecules act as versatile scaffolds or guides, leveraging interactions with DNA, RNA and proteins to influence gene expression through highly diverse, and context-dependent pathways [251]. This regulatory complexity is further compounded by their multiple splice variants, dynamic subcellular localisation, low expression levels and variable protein partners [251]. Although a few proteomic studies have examined lncRNA interactors during VSMC differentiation [296], studying these interactions remains difficult. Methodological limitations, including the difficulty of capturing RNA-mediated complexes and the unreliability of simple overexpression studies, hinder the full elucidation of their molecular mechanisms [297].

#### 3.4.3. Circular RNAs

CircRNAs are a highly stable class of ncRNA that are produced through the retro-splicing of pre-mRNAs, resulting in a covalently closed circular structure [298]. This unique structure renders them resistant to polyA-dependent degradation and enables them to perform a variety of biological functions, such as regulating cardiovascular development and disease progression [298,299,300]. CircRNAs regulate gene expression by acting as molecular scaffolds that cooperate with RNA-binding proteins and regulate transcription/translation. Notably, they also act as “sponges” that sequester miRNAs [298,301]. Furthermore, some circRNAs can be translated to produce proteins, sharing many mechanisms with linear lncRNAs [298,301].

Several circRNAs are key regulators in VSMC biology. For example, circANRIL, which is derived from the CAD-risk 9p21 locus, is considered atheroprotective because it inhibits VSMC proliferation, thereby opposing the function of its linear counterpart [302,303]. Similarly, *circ_Lrp6* promotes VSMC proliferation and suppresses differentiation by sponging miR-145, and its silencing in vivo reduces neointimal hyperplasia [304]. Another circRNA, *circ0000006* is upregulated in aortic dissection (AD), where it sponges miR-483-5p to enhance KDM2B expression, driving the PDGF-BB-mediated phenotypic switch [305]. circRNAs can also be packaged into exosomes, mediating interactions between ECs and VSMCs to modulate VSMC phenotype [304], as was previously shown for miRNAs [306].

The therapeutic potential of circRNAs is growing rapidly due to their stability and regulatory power, positioning them as a promising approach for targeting miRNA dysregulation [307]. However, two main challenges hinder clinical translation. Firstly, safe, cell-specific and lesion-targeted delivery systems are needed to effectively harness their regulation and avoid paradoxical outcomes. Secondly, their complex, context-dependent function requires further clarification of their mechanisms, particularly with regard to their non-sponging roles.

### 3.5. RNA Modifications

Most types of RNA, both coding and non-coding, can undergo modification [308]. Although tRNA and rRNA modifications are abundant and play a key role in providing structure and function to host RNAs, this review focuses on modifications in mRNA and lncRNA. Readers interested in tRNA and rRNA are directed to consult other reviews [309,310].

The epitranscriptome of mRNA is diverse and continues to expand as detection techniques improve [311]. This review focuses on the most prevalent and well-characterised modifications, including N6-methyladenosine (m6A), pseudouridine (ψ), N5-methylcytosine (m5C), N1-methyladenosine (m1A) and adenosine-to-inosine (A-to-I) editing [308]. Other modifications exist, but they are present on mRNA only in limited quantities [312]. The most prevalent and well-characterised internal modification of eukaryotic mRNA is m6A [312]. Similarly to those of other epigenetic marks, the deposition, removal and recognition of m6A are performed by three major sets of proteins: the “writers”, “erasers” and “readers”, respectively [312]. In most eukaryotes, the core writer complex, which includes the catalytically active METTL3 and the structural METTL14 [313], deposits m6A by interacting with the cofactor S-adenosylmethionine (SAM) and regulatory subunits such as WTAP and VIRMA [314]. Removal is catalysed by erasers such as the fat mass and obesity-associated protein (FTO) and alkB homologue 5 (ALKBH5) [313]. The m6A mark exerts its biological effects by recruiting diverse “reader” proteins. The most well-characterised of these are the YTH family proteins (YTHDF1-3 and YTHDC1-2), which recognise m6A within a RRACH motif [313,315]. The primary role of YTHDF proteins is to decrease mRNA stability by targeting transcripts for decay; however, their functions are diverse and they can also influence translation and splicing [316,317,318,319,320,321]. YTHDC1 is associated with alternative splicing and the control of nuclear export [322,323], while YTHDC2 influences translation and mRNA stability [324]. Other readers include proteins in the insulin-like growth factor 2 mRNA-binding protein 2 (IGF2BP2), heterogeneous nuclear ribonucleoprotein (HNRNP) and eukaryotic translation initiation factor 3 (eIF3) families, which affect stability, splicing and translation [325,326,327].

The biological effects of m6A readers, particularly those of the YTHDF family, involve several mechanisms. These include the recruitment of the CCR4-NOT deadenylation complex [328], the recruitment of RNase P/MRP to promote endoribonucleolytic cleavage [329] and the interaction with UPF1 to promote decapping [330]. Another recently proposed mechanism is CDS-mediated decay (CMD), whereby the m6A group impedes normal codon decoding during translation. This leads to ribosome stalling and mRNA degradation in a YTHDF2-dependent manner [331]. By influencing key aspects of RNA metabolism, including splicing, nuclear export, translation and stability, m6A is involved in fundamental cellular processes, such as differentiation, proliferation, apoptosis and stem cell renewal [332,333,334,335]. Building on this foundational knowledge, recent studies have begun to elucidate the role of m6A in complex pathologies, including CVD [336].

The effects of m6A in VSMCs are multifaceted and context-dependent, exhibiting both pro-pathogenic and protective/homeostatic roles [337]. In intimal hyperplasia, METTL3 and YTHDF3 promote a VSMC phenotypic switch by enhancing *Profilin 1* translation, thereby facilitating the contribution of VSMCs to atherosclerosis through increased *MRTFA* translational efficiency [338,339]. Additionally, YTHDF2 promotes VSMC proliferation in pulmonary vascular remodelling by increasing *Myadm* stability [340]. Consistently, we have documented that YTHDF reader proteins undergo autophagic regulation during cell-to-cell contact-dependent cell cycle exit in non-cancerous cells [341]. Furthermore, the dramatic post-translational decrease in YTHDF2 during VSMC redifferentiation, which is induced by cell-to-cell contact, directly impacts the expression of the lncRNA *DAGARR* [296].

Conversely, homeostatic or protective roles have also been reported. For example, METTL3 maintains vascular contraction by stabilising the mRNA of contraction-related genes such as *Myh11* [342] and YTHDF2 can protect VSMCs from osteogenic differentiation by downregulating *RUNX2* [343,344]. Furthermore, increased FTO expression exacerbates the harmful VSMC phenotypic switch by promoting *KLF5* expression in AA [345] and YTHDF1 activity can prevent neointima formation in response to injury [346].

The conflicting results across studies likely stem not only from differential expression of m6A regulators and the state-dependent availability of their RNA substrates. Since phenotypic switching fundamentally reprograms the VSMC transcriptome, the pool of transcripts available for methylation shifts dramatically between contractile and synthetic states. Consequently, the outcome of perturbation of the m6A machinery is dictated by the underlying identity of the cell. For example, writers like METTL3 may stabilise homeostatic transcripts in quiescent cells but can also enhance drivers of calcification in injured vessels. Furthermore, as several m6A readers compete for the same substrates, the relative abundance of these proteins will ultimately determine the fate of modified mRNAs. Therefore m6A regulators should be viewed as context-dependent modulators whose function is conditioned by the transcriptional landscape, rather than as intrinsic atherogenic or protective factors.

Although the characterisation of other modifications lags behind that of m6A, reports linking them to vascular biology are starting to emerge. For example, m1A is dysregulated in ischaemic stroke [347], while the m5C writer NSUN2 prevents PDGF-BB-induced SMC proliferation, migration and inflammation [348] and plays a role in hypoxia-induced vascular remodelling via *circCCNL2* [349].

The enzyme ADAR1 is responsible for deaminating adenosine to inosine (A-to-I editing), and it suppresses the inflammatory response to immunogenic dsRNA [350]. ADAR1 deficiencies cause severe phenotypes, including embryonic lethality in mice and Aicardi–Goutières syndrome (AGS) in humans [350]. Studies have revealed conflicting roles for ADAR1 in VSMCs: Heterozygous ADAR1 deletion has been shown to enhance VSMC phenotypic modulation in AAA and vascular remodelling, indicating a potential harmful effect [351]. Conversely, VSMC-specific ADAR1 deficiency can cause vascular structural alterations and apoptosis, leading to lethality [352]. Furthermore, ADAR1 protects VSMCs by avoiding the activation of the dsRNA sensor MDA5. This promotes vascular integrity and limits atherosclerosis, calcification and PH [353,354]. These contradictions highlight the importance of understanding the highly cell- and time-specific roles of RNA modifications in order to identify appropriate windows for targeted treatment.

In conclusion, the m6A-anchored epitranscriptome plays a crucial role in regulating VSMC phenotype and pathology. However, translating this into therapeutics faces significant challenges. Firstly, the mechanistic complexity is immense, as the function of a single modification is often contradictory and context-dependent, determined by the specific ‘reader’ proteins recruited. This ambiguity is further compounded for less well-characterised marks, such as m5C and A-to-I editing, where high-resolution mapping remains limited. Secondly, the deep interconnectivity with other epigenetic layers (e.g., m6A regulating lncRNA *DAGARR*) necessitates a comprehensive, multi-omics approach. Future research must prioritise high-resolution and cell-specific mapping, as well as unravelling the intricate ‘reader code’ in order to safely realise the therapeutic potential of RNA modifications in vascular disease. The advent of long-read sequencing, which has the potential to detect multiple modifications in the same RNA molecule [355], will also enable researchers to study the complex interactions that exist between different RNA modifications.

It is worth noting that the different epigenetic mechanisms outlined in this review build on each other, with each new layer potentially interacting with the others. Examples include RNA modifications deposited in lncRNAs [296], RNA editing of miRNAs [356], m6A-regulated expression of histone modifiers [357] and lncRNA acting as a scaffold for histone modifier complexes [358].

## 4. Perspectives

To develop targeted therapies that go beyond generalised cellular depletion, it is crucial to understand the cellular origins and fates of cells within a vascular lesion. For decades, the contribution of different cell types to intimal hyperplasia has been a significant point of controversy. While single-cell lineage tracing has provided valuable insight into the origin and fate of VSMCs in vascular lesions, it often lacks the temporal and molecular resolution necessary to identify the precise cellular events or signalling cascades that precede or drive these intricate changes. A transformative advancement would be the application of inducible double lineage tracing. This system offers unparalleled resolution for analysing the intricate signalling pathways involved in a specific differentiation trajectory. Therefore, a significant advance would be to apply these systems to exhaustively identify the molecular pathways involved in establishing specific VSMC phenotypes. For example, one could design a system that labels all contractile VSMCs at the start of a disease model and then uses a second reporter to permanently mark cells that activate a ‘synthetic’ phenotype marker at a later stage. Professor Owens’s laboratory has already pioneered this strategy [147]. Combining double lineage tracing with the knockout of specific genes would provide valuable insight into the true importance of a given molecule and its role in the resulting VSMC phenotype during disease progression.

The therapeutic approach to vascular diseases is shifting from non-specific VSMC depletion strategies to interventions that aim to preserve or restore beneficial VSMC phenotypes within the lesion. Non-selective VSMC loss has been shown to compromise plaque stability and encourage the invasion of the neointima by other cell types, such as macrophages and ECs. This contributes to disease progression [359]. As this review has established, epigenetic regulation plays a central role in controlling VSMC phenotype and function. Pathological conditions such as disturbed haemodynamic forces and oxidative stress can disrupt this programme, leading to maladaptive transitions in phenotype that contribute to vascular remodelling. In this context, epidrugs, a class of therapeutic agents that target the epigenome to influence gene expression and cell state, are emerging as a promising strategy for preventing or reversing VSMC phenotypes [360]. Several classes of epidrugs are currently in preclinical and clinical development for CVD. These include DNMT inhibitors, such as decitabine; HDAC inhibitors, such as SAHA and valproic acid; EZH2 inhibitors, such as tazemetostat and GSK126; and BRD4 inhibitors, such as the BET inhibitor JQ1 [361]. Other epidrugs include SIRT1/3 activators, such as resveratrol and NAD+ precursors, which restore deacetylase activity and redox balance [362,363,364], and metabolic cofactors, such as α-ketoglutarate and SAM donors, which normalise enzymatic methylation/demethylation activity [203,365,366]. These interventions have been shown to be effective in preclinical and clinical models by reversing the hyperproliferative and anti-apoptotic phenotypes of vascular cells [360,361].

Despite the therapeutic promise of epidrugs, a major challenge remains. The lack of specificity of many of the epidrugs that are currently available, such as HDAC and DNMT inhibitors, can result in off-target and systemic side effects. To address this issue, an innovative approach has emerged in the form of proteolysis-targeting chimaeras (PROTACs), which selectively degrade target proteins via the ubiquitin–proteasome system [367]. Unlike conventional inhibitors, which block catalytic sites, PROTACs eliminate target proteins entirely, enabling potent modulation of the key effects implicated in epigenetic and epitranscriptomic dysregulation [367]. Another promising approach to achieving treatment specificity is CRISPR-CAS9 gene editing, which has long been proposed as a treatment for dyslipidemias that cause atherosclerosis. This approach has recently completed a phase 1 trial that lowered ANGPTL3 levels [368], mimicking the protective effect of naturally occurring loss-of-function mutations against coronary artery disease [369]. Moving forward, more research will be needed to expand this technology beyond monogenic CVDs, while simultaneously minimising the risk of off-target effects.

The ongoing discovery of novel epigenetic and epitranscriptomic mechanisms continues to reveal new therapeutic targets opportunities. One particularly promising area is m6A RNA modification. Small molecule inhibitors targeting the “writers” and “readers” of m6A are already being developed for use in cancer treatments. Notably, the first METTL3 inhibitor has successfully completed Phase I of a clinical trial for solid tumours (NCT05584111, NCT06975293) and demonstrated favourable safety and efficacy profiles. It has now advanced to Phase Ib/II [370]. In light of recent reports implicating METTL3 in atherosclerosis and vascular remodelling [339,371,372], it is plausible that similar inhibitory strategies could be adapted for CVD. The current dominance of m6A-targeted therapies likely reflects the fact that this is the most prevalent and well-characterised RNA modification. We anticipate that therapeutic targets will expand in the future as more mechanisms involving other RNA modifications are elucidated. Advances in epitranscriptomics have also enabled the development of mRNA therapies. Understanding how RNA modifications modulate immune recognition of exogenous RNA has been pivotal to the success of modern mRNA vaccine platforms, which now form the basis of therapeutic development for cancer [373] and CVDs [374]. In preclinical models, delivering IL-10 mRNA to atherosclerotic lesions using nanoparticles has been shown to reduce inflammation, decrease necrotic core and increase fibrous cap thickness [375]. Furthermore, initial clinical trials involving the use of naked *VEGF-A* mRNA in patients undergoing coronary artery bypass grafting have yielded favourable results [376]. Beyond mRNA therapies, siRNA- and miRNA-based approaches are being actively explored for cardiovascular interventions. Recently, a preclinical study reported that BHF7 siRNA is a potent therapeutic for preventing coronary artery bypass graft failure [377]. This therapy targets the lncRNA *SMILR*, a key driver of vascular VSMC proliferation [378]. BHF7 has been shown to effectively silence *SMILR* expression in both in vitro human VSMCs and ex vivo human saphenous vein tissue, thereby blocking VSMC proliferation and downregulating pro-proliferative genes [377]. Importantly, the authors reported that BHF7 showed no significant cytotoxicity or interferon response and could be delivered to the graft within the 30 min clinical surgery window. These findings supports the development of BHF7 as an ex vivo RNA therapeutic that can be applied directly to the graft [377].

The advancement of these therapeutic strategies is inherently linked to the development of robust biomarkers. Circulating ncRNAs, particularly miRNAs, are emerging as promising non-invasive biomarkers for cardiovascular risk stratification, diagnosis and prognosis, thanks to their stability and accessibility [379]. However, there is also a critical need for localised, cell-specific biomarkers that can accurately capture pathological VSMC transitions and guide personalised interventions, such as those related to oxidative stress, inflammation and plaque stability [380]. Furthermore, future therapeutic development must address the underrepresentation of patient populations, particularly women, in CVD research and clinical trials [381].

As high-resolution lineage tracing and advanced omics technologies, such as spatial and single-cell transcriptomics and proteomics, continue to reveal the molecular mechanisms that govern the phenotypic control in VSMCs, the development of highly specific epidrugs, such as PROTACs and RNA-based therapies, is expected to transform the prevention and treatment of vascular diseases by targeting maladaptive VSMC phenotypes.

## 5. Conclusions

VSMC phenotypic plasticity is a key mechanism in the development and progression of major CVDs. The transition from a quiescent, contractile phenotype to a proliferative, synthetic state is governed by a complex, multilayered epigenetic network involving DNA methylation, histone modifications, chromatin remodelling, ncRNAs and RNA modifications. These mechanisms integrate mechanical, metabolic and microenvironmental cues, often converging on the MYOCD-SRF transcriptional axis. The reversible nature of these epigenetic alterations provides a powerful therapeutic window through which vascular homeostasis can be restored by selectively inhibiting histone deacetylase isoforms or specific ncRNAs.

However, translating these insights into effective clinical treatments remains challenging. Researchers must design interventions that can selectively inhibit pathological VSMC functions, such as proliferation and calcification, while preserving protective functions such as fibrous cap formation. Furthermore, achieving precise, cell-specific delivery within the vascular wall poses a significant obstacle to therapeutic success.

Therefore, future research should aim to comprehensively map the epigenetic regulatory circuits that govern VSMC fate and develop advanced delivery methods that can safely and efficiently target diseased cells. The VSMC phenotype ultimately arises from an integrated network of mechanical, redox and metabolic cues that remodel chromatin and gene expression. This dynamic interplay generates an epigenetic memory of haemodynamic stress that perpetuates vascular remodelling. Targeting this mechanosensitive–redox–epigenetic axis is a promising approach for restoring vascular plasticity and halting the progression of chronic vascular disease.

## Data Availability

No new data were created or analyzed in this study. Data sharing is not applicable to this article.

## Abbreviations

The following abbreviations are used in this manuscript:

AA	Aortic Aneurysm
AAA	Abdominal Aortic Aneurysm
ACTA 2	Alpha Smooth Muscle Actin Gene
AD	Aortic Dissection
Ang II	Angiotensin II
AVP	Vasopresin
BMPR2	Bone Morphogenetic Protein Receptor Type 2
CAD	Coronary Artery Disease
CBP	CREB Binding Protein
CMA/CSVD	Cerebral Microangiopathy/Cerebral Small-Vessel Disease
CNN1	Calponin 1
CVD	Cardiovascular Disease
DES	Drug-Eluting Stent
DNA	Deoxyribonucleic Acid
DNMT	DNA Methyltransferase
EC	Endothelial Cell
ECM	Extracellular Matrix
EndMT	Endothelial-to-Mesenchymal Transition
ESC	Embryonic Stem Cell
ET-1	Endothelin-1
FA/FAK	Focal Adhesion/Focal Adhesion Kinase
FBN1	Fibrillin-1
GLI1	Glioma-Associated Oncogene Homologue 1
H3K4me1/2/3	Histone H3 Lysine 4 Mono/Di/Tri-Methylation
H3K9me3/H3K27me3	Histone H3 Lysine 9/27 Trimethylation
HAT/HDAC	Histone Acetyltransferase/Histone Deacetylase
HDM/HMT	Histone Demethylase/Histone Methyltransferase
HIF-1α	Hypoxia-Inducible Factor 1 Alpha
IHD	Ischemic Heart Disease
IGF1	Insulin-like Growth Factor 1
IST	In-Stent Restenosis
iPSC	Induced Pluripotent Stem Cell
KLF4	Kruppel-Like Factor 4
Lgals3	Galectin-3 Gene
LINC	Linker of Nucleoskeleton and Cytoskeleton
MAPK	Mitogen-Activated Protein Kinase
MBD	Methyl-CpG Binding Domain
MECP2	Methyl-CpG Binding Protein 2
MFS	Marfan Syndrome
MRTF-A/B	Myocardin-Related Transcription Factor A/B
mTOR/mTORC1	Mammalian Target of Rapamycin/Complex 1
MYOCD	Myocardin
MYH11	Myosin Heavy Chain 11
NAD^+^	Nicotinamide Adenine Dinucleotide (Oxidised form)
NF-κB	Nuclear Factor Kappa B
NOX/ROS	NADPH Oxidase/Reactive Oxygen Species
PAH/PH	Pulmonary Arterial Hypertension/Pulmonary Hypertension
PAD	Peripheral Arterial Disease
PDGF	Platelet-Derived Growth Factor
PDK/PDH	Pyruvate Dehydrogenase Kinase/Pyruvate Dehydrogenase
PI3K/AKT	Phosphoinositide 3-Kinase/Protein Kinase B
PKCα	Protein Kinase C Alpha
PRC2	Polycomb Repressive Complex 2
PROTAC	Proteolysis Targeting Chimaera
PVR	Pulmonary Vascular Resistance
RA/RAR/RXR	Retinoic Acid/Retinoic Acid Receptor/Retinoic X Receptor
RAS	Renin Angiotensin System
ROS	Reactive Oxygen Species
Sca-1	Stem Cell Antigen-1
SIRT	Sirtuin
α-SMA	Smooth Muscle Alpha-Actin
SMC/VSMC	Smooth Muscle Cell/Vascular Smooth Muscle Cell
SMMHC	Smooth Muscle Myosin Heavy Chain
SRF	Serum Response Factor
SUV39H1	Suppressor of Variegation 3-9 Homologue 1
TAZ	Transcriptional Coactivator with PDZ-Binding Motif
TET/5mC/5hmC/5fC/5caC	Ten-Eleven Translocation Enzyme/5-Methylcytosine/5-Hydroxymethylcytosine/5-Forylcytosine/5-Carboxycytosine
TGF-β	Transforming Growth Factor Beta
TNFα	Tumour Necrosis Factor Alpha
VGS	Vein Graft Stenosis
YAP	Yes-Associated Protein
